# Carbon Black Particle Exhibits Size Dependent Toxicity in Human Monocytes

**DOI:** 10.1155/2014/827019

**Published:** 2014-02-05

**Authors:** Devashri Sahu, G. M. Kannan, R. Vijayaraghavan

**Affiliations:** ^1^Pharmacology and Toxicology Division, Defence Research and Development Establishment, Jhansi Road, Gwalior 474002, India; ^2^Saveetha University, P.H. Road, Chennai 600077, India

## Abstract

Increased levels of particulate air pollution are associated with increased respiratory and cardiovascular mortality and morbidity. Some epidemiologic and toxicological researches suggest ultrafine particles (<100 nm) to be more harmful per unit mass than larger particles. In the present study, the effect of particle size (nano and micro) of carbon black (CB) particle on viability, phagocytosis, cytokine induction, and DNA damage in human monocytes, THP-1 cells, was analysed. The cells were incubated with nanosize (~50 nm) and micron (~500 nm) size of CB particles in a concentration range of 50–800 *µ*g/mL. The parameters like MTT assay, phagocytosis assay, ELISA, gene expression, and DNA analysis were studied. Exposure to nano- and micron-sized CB particles showed size- and concentration dependent decrease in cell viability and significant increase in proinflammatory cytokines IL-1**β**, TNF-**α** and IL-6 as well as chemokine IL-8 release. Gene expression study showed upregulation of monocyte chemoattractant protein-1 gene while cyclooxygenase-2 gene remained unaffected. Nano CB particles altered the phagocytic capacity of monocytes although micron CB had no significant effect. CB particles did not show any significant effect on DNA of monocytes. The investigations indicate that CB particles in nanosize exhibit higher propensity of inducing cytotoxicity, inflammation, and altered phagocytosis in human monocytes than their micron size.

## 1. Introduction

The nanoparticle industry has expanded substantially in recent years leading to exposure of various nanomaterials to human and environment. Particle size plays an important role in determining the particular biological behavior of nanomaterials. Due to their extreme small size, nanoparticles possess specific large surface area, which makes the number of surface atoms or molecules increasing exponentially. Hence, particles at nanorange exhibit much higher chemical and biological reactivity than fine particles [1]. The risks associated with nanoparticles exposure require investigation due to evidence that these particles can be more inflammogenic and toxic than larger particles comprising of the same material [2]. In recent years, size-dependent toxicity between micro- and nanoscale particles has been demonstrated [3–5].

Carbon black (CB) has wide industrial applications, and used as a reinforcement agent in rubber products, black pigment in printing inks and lithography, electrode for batteries in electrical conductors and during the finishing process of leather goods production. Additionally, carbonaceous nanoparticles are present as an environmental contaminant. Combustion processes are a significant source of carbon nanoparticles. Elemental carbon-based nanoparticles with a diameter of less than 100 nm are a major part of diesel exhaust and ambient pollution. After deposition in the lungs, larger particles are phagocytized by alveolar and airway macrophages [6, 7], but the fine and ultrafine carbon particles remain in the lungs for a longer period of time [8]. Ultrafine particles are phagocytized to a minor extend but they can still enter macrophages and epithelial cells and even penetrate into the circulation. Thus, ultrafine particles not only trigger local inflammatory reactions in the lungs but also cause systemic extrapulmonary effects [9]. Ultrafine particles also have the capacity to inhibit phagocytosis by alveolar macrophages [10]. Macrophages and their monocyte progenitors are major elements of the inflammatory response. In addition to performing phagocytosis, they can release inflammatory mediators such as cytokines and chemokines, crucially involved in destruction of microbes and particles using various enzymatic systems [11]. CB nanoparticles are reported to cause cytotoxic injury, increase levels of proinflammatory chemokines, and inhibit cell growth [12]. Epidemiological as well as experimental studies have confirmed the role of CB nanoparticles in aggravating pulmonary disorders such as asthma, lung cancer, pulmonary fibrosis, and systemic cardiovascular disorders [13].

In this study, CB particles were chosen considering their production in huge quantities posing high environmental risk compromising health of general population [13, 14]. Because of the sporadic information on in vitro size-dependent effect of CB particles, the present study was conducted to determine the effect of their nano- and micron-sized particles on viability, phagocytosis, and cytokine induction in human monocytes, THP-1 cells. These undifferentiated cells express many of the properties of monocytes and represent a model of innate immune system [15]. These cells are an essential link between the adaptive and innate immune responses because they develop into various forms of antigen-presenting cells (macrophages and dendritic cells). They are often used as a model to study human inflammatory responses, which allow for the possibility of elucidating the interactions of nanoparticles with innate immune cells [16, 17].

## 2. Materials and Methods 

### 2.1. Particle Preparation and Characterization

Carbon nanopowder <50 nm and carbon powder ~500 nm were purchased from Sigma-Aldrich. Physicochemical properties of particles were analyzed using transmission electron microscopy (TEM), dynamic light scattering (DLS), and zeta potential analyzer. The morphology and size of particles in the stock dispersion were determined by TEM. Dry powder of particles was suspended in cell culture medium at a concentration of 1 mg/mL and then sonicated at room temperature for 10 minutes to form a homogeneous suspension. After sonication and stabilization, the TEM samples were prepared by drop coating of the stock suspension on carbon-coated copper grids. The films on the grids were allowed to dry prior to measurement. TEM measurements were performed at an accelerating voltage of 120 kV (Model 1200EX, JEOL Ltd., Tokyo, Japan). ZetaPALS (Brookhaven Instruments Corporation, Holtsville, NY) was used to determine the hydrodynamic size and zeta potential of particle suspension in cell culture medium.

### 2.2. Cell Culture

The human monocytic cell line, THP-1 was obtained from National Centre for Cell Sciences, Pune, India. They were maintained in RPMI 1640 medium supplemented with 10% heat inactivated fetal bovine serum (FBS), L-glutamine (2 mM), streptomycin (100 *μ*g/mL), and penicillin (100 U/mL). Cells were cultured at 37°C in a humidified atmosphere containing 5% CO_2_. THP-1, cells were seeded into 24-well plates at 1 × 10^6^ cells/mL and exposed to particles in the concentration range of 50 to 800 *μ*g/mL for 24 h. Based on the results of screening study (data not shown) done with different concentrations of particles and previous reports [18, 19], a concentration range was selected. The cells were exposed within 10 min of preparation of nano- and micro-CB suspensions. Cell-free controls were included in order to assess the interference of particles with each assay.

### 2.3. Assessment of Cytotoxicity

THP-1 cells were incubated with CB particles (nano and micro) in a concentration range of 50, 100, 200, 400, and 800 *μ*g/mL for 24 h. Following this incubation period, cytotoxicity of particles was assessed using MTT assay [20]. Briefly, MTT (20 *μ*L per well of 5 mg/mL stock) was added and incubated for 4 h. Supernatants were removed by centrifugation and then 300 *μ*L of DMSO was added. After thorough mixing, optical density at 570 nm was detected by microplate reader (BioTek, USA). Control values (without stimuli) were set at 100% viable and all values were expressed as a percentage of the control and respective TC-50 (particle concentration inducing 50% cell mortality) concentrations were calculated using GraphPad Prism software.

### 2.4. Phagocytosis Assay

The phagocytic ability of THP-1 cells after 4 h exposure to varying concentrations of CB particles (namely, 1/2TC,50, TC50 and 2TC50) was assessed by measuring their ability to phagocytose 1 *μ*m latex beads (1 *μ*m Latex beads, carboxylate modified polystyrene, and fluorescent yellow-green). The method used was modified method of Schroeder and Kinden [21]. After exposure, cells were washed two times with PBS to remove excess particles. Culture medium containing latex beads at a bead-to-cell ratio of 10 : 1 was transferred to the culture wells. Monocyte and bead suspensions were then incubated for 1 h to allow phagocytosis. Beads not phagocytosed were removed by centrifugation at 225 g for 5 min; the cell pellet was then resuspended in phosphate buffer saline. The process was repeated three times and finally the cells were vortexed for 10 s and fluorescence of the cells was determined at an excitation and emission wavelengths set at 440 and 485 nm, respectively. Cell viability during phagocytosis assay was monitored by trypan blue exclusion. Viability was 95 ± 5% throughout the assay. Microscopic images of phagocytosed latex beads, only at one concentration, that is, TC-50 concentration of test particles, were taken. After washing, the cells were seen under microscope. DIC images or paired DIC and fluorescence images of phagocytosed beads by monocytes were acquired using fluorescent microscope (Zeiss, Germany) with 40x dry and 100x (oil immersion) objectives.

### 2.5. Cytokine Analysis

To investigate the effect of CB particles on cytokine production, an enzyme linked immunosorbent assay (ELISA) was performed. For determination of IL-1*β*, IL-6, TNF-*α*, and IL-8, monocytes were cultured at 1 × 10^6^ cells/mL and were exposed to TC-50 concentration of CB particles (nano carbon: 591.4 *μ*g/mL and micro carbon 687.1 *μ*g/mL) for 6, 18, 24, and 48 h. After particle exposure, cell-free supernatants were harvested via successive 10 min centrifugations (2,000 rpm, 7,000 rpm, and 13,000 rpm) and stored at −80°C until analysis. ELISA was performed according to the manufacturer's protocol (Abcam ELISA Kit) and absorbance values were measured using microplate reader (BioTek Instruments, USA). A minimum of three independent experiments were performed and concentrations calculated from the linear regression equation were derived from a set of standard absorbance values.

### 2.6. Gene Expression Analysis

Gene expression analysis of COX-2 and MCP-1 was evaluated by exposing cells to TC-50 concentration of CB particles (nano carbon: 591.4 *μ*g/mL and micro carbon 687.1 *μ*g/mL) for 24 h. Total RNA were isolated using RNeasy Mini Kit (Qiagen, USA). The concentration and integrity of RNA was measured using multimode microplate reader (BioTek, USA) prior to the experiment. The Enhanced Avian HS RT-PCR kit (Sigma, USA) was used for the amplification of COX-2, MCP-1, and 18 sRNA gene, according to the manufacturer's instructions. Amplified cDNA products were separated on 1.2% agarose gel by electrophoresis. The primer sequences of amplified genes were shown in Table 1.

### 2.7. Detection of DNA Damage

THP-1 cells exposed to nano- and microparticles of CB (0, 50, 100, 200, 400, and 800 *μ*g/mL) for 24 h were collected into tubes and washed with PBS. The cells were incubated for 3 h in lysis buffer (20 mM Tris-HCl, pH 8.0, 5 mM EDTA, 0.1 M NaCl, 0.5% SDS, and 100 *μ*g/mL RNase) at 37°C. After incubation, phenol : chloroform (1 : 1) mixture was used to extract DNA. By adding an equal volume of ice-cold absolute isopropanol, DNA was precipitated. DNA was dissolved in 50 *μ*L of 1X TE (10 mM Tris, 1 mM EDTA, and pH 8.0) buffer. 20 *μ*g of DNA was loaded onto 1.2% agarose gel and electrophoresis was carried out at 60 V for 2 h with TBE as the running buffer. DNA in the gel was visualized under UV light [22].

### 2.8. Statistical Analysis

Statistical analysis was carried out with GraphPad Prism 4 statistical software (Graphpad Software Inc., CA, USA). One-way analysis of variance (ANOVA) with Tukey's method for multiple comparisons was used to evaluate the various responses induced by different concentrations of particles and statistical comparisons between particle sizes were performed with two-way ANOVA, followed by a Bonferroni posttest. Differences were considered statistically significant when the *P* value was less than 0.05.

## 3. Results

### 3.1. Particle Characterization

**Figure 1:**
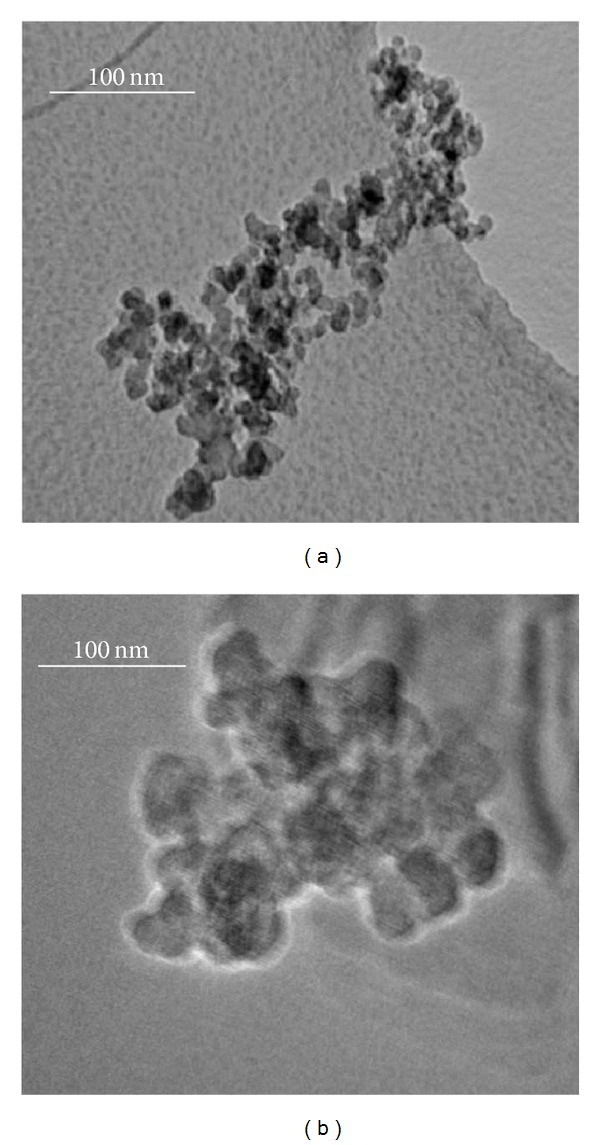
Transmission electron micrograph of carbon particles: (a) nanocarbon and (b) microcarbon. Scale bar is 100 nm.

TEM analysis was performed to determine the morphology and size of the particle and micrographs are shown in Figure 1. The particles were found nearly spherical and cubical in shape. The primary sizes of the particles estimated from TEM images (Figure 1) were presented in Table 2. Since nanoparticles often form agglomerates in a solution, the hydrodynamic sizes of the dispersed particles and their agglomerates in cell culture medium were estimated using ZetaPALS. These values were found larger than the *per se* particle size measured by TEM (Table 2).

### 3.2. Cell Viability

**Figure 2:**
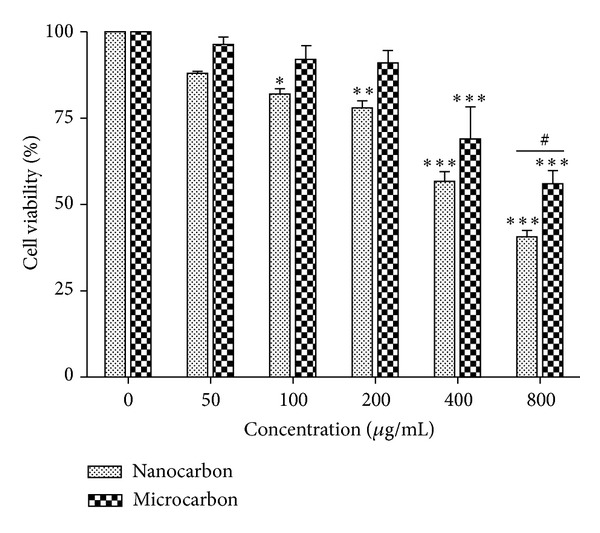
Percentage of the metabolic activity of THP-1 cells upon exposure to varying concentrations of carbon particles, as determined by the MTT assay. Cells were exposed to 50, 100, 200, 400 and 800 *μ*g/mL of nano- and microcarbon particles for 24 h. The results are expressed as a percentage relative to controls and are presented as mean ± SEM of three independent replicate experiments. Significance is indicated by *P < 0.05, **P < 0.01 and ***P < 0.001, versus control. ^#^ *P* < 0.05 indicates significant difference between the particle sizes.

After 24 h exposure of THP-1 cells to varying doses (50, 100, 200, 400, and 800 *μ*g/mL) of nano- and micro-CB particles, cellular metabolic activity was detected by MTT assay. The cell viability decreased in a concentration- and size-dependent manner following exposure to CB particle (Figure 2) and a significant decrease was observed at concentration from 100 to 800 *μ*g/mL for nano carbon particles and at 400 and 800 *μ*g/mL for micro carbon particles. The nano carbon particles decreased the percentage of cell viability from 82% to 41% while micro carbon particles decreased it from 69% to 56%. The significant size selective difference (*P* < 0.01) between nano- and micro carbon was found only at highest concentration of 800 *μ*g/mL. The TC-50 value (particle concentration causing 50% cell mortality) calculated for nano- and micro carbon was found to be 591.4 *μ*g/mL and 687.1 *μ*g/mL, respectively (Table 3).

### 3.3. Phagocytic Capacity of THP-1 Cells

**Figure 3:**
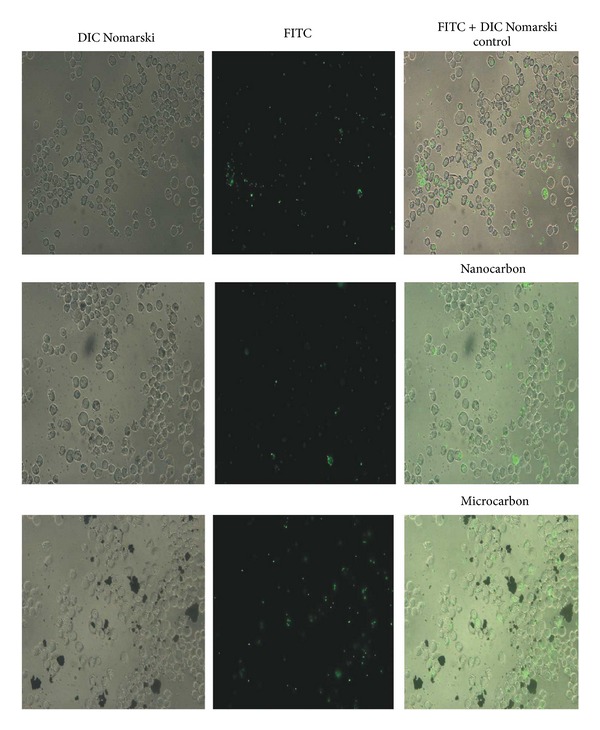
Photomicrographs of THP-1 cells phagocytosing latex beads. THP-1 cells were incubated with TC-50 concentration of carbon particles (nano carbon: 591.4 *μ*g/mL and micro carbon 687.1 *μ*g/mL) for 4 h; then, latex beads were allowed to phagocytose by THP-1 cells for 1 h. After washing, fluorescent images were taken (magnification 400x).

**Figure 4:**
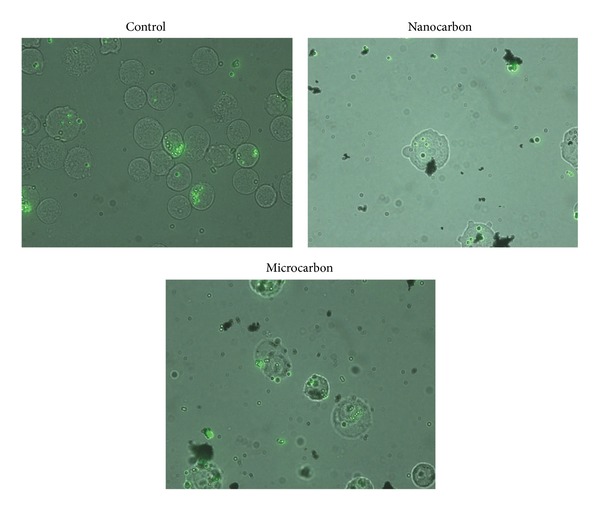
Representative photomicrographs of THP-1 cells phagocytosing latex beads (magnification 1000x).

**Figure 5:**
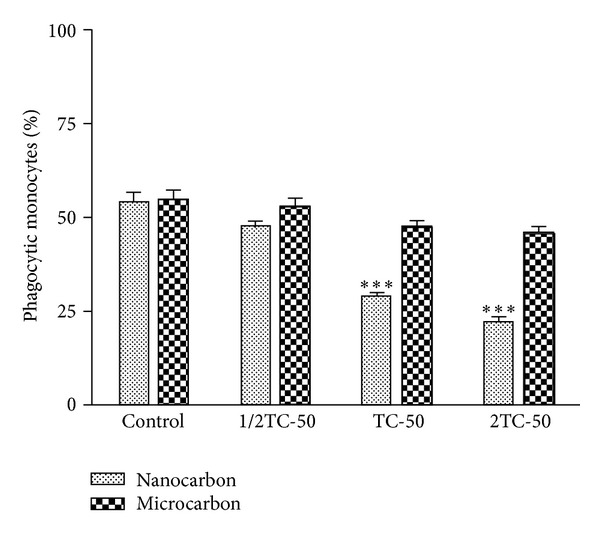
Percentage of cells capable of phagocytosing the indicator latex beads after the uptake of test particles (i.e., phagocytic monocytes). Cells were exposed to 1/2TC-50, TC-50, and 2TC-50 concentrations of nano carbon (295.7 *μ*g/mL, 591.4 *μ*g/mL, and 1182.8 *μ*g/mL, resp.) and micro carbon (343.5 *μ*g/mL, 687.1 *μ*g/mL and 1374.2 *μ*g/mL, resp.) for 4 h. The results are expressed as a percentage relative to controls and are presented as mean ± SEM of three independent replicate experiments. Significance is indicated by **P < 0.01 versus control.

The phagocytic ability of the monocytes was measured after the uptake of test particles. The assay was carried out by taking 3 different concentrations of test particle, namely, 1/2 TC-50, TC-50, and 2TC-50 for each particle. A significant reduction in the phagocytosis of indicator latex beads occurred after exposure to all concentrations of nano-CB particles (*P* < 0.01), while no significant reduction was observed with their microsize (Figure 3). Fluorescent images represent monocytes phagocytosing latex beads (Figure 4). Qualitatively control cells were found to phagocytose more beads as compared to nano-CB particle exposed monocytes whereas the phagocytic capacity of monocytes was not affected by the presence of micro-CB particles (Figure 5).

### 3.4. Proinflammatory Cytokine

**Figure 6:**
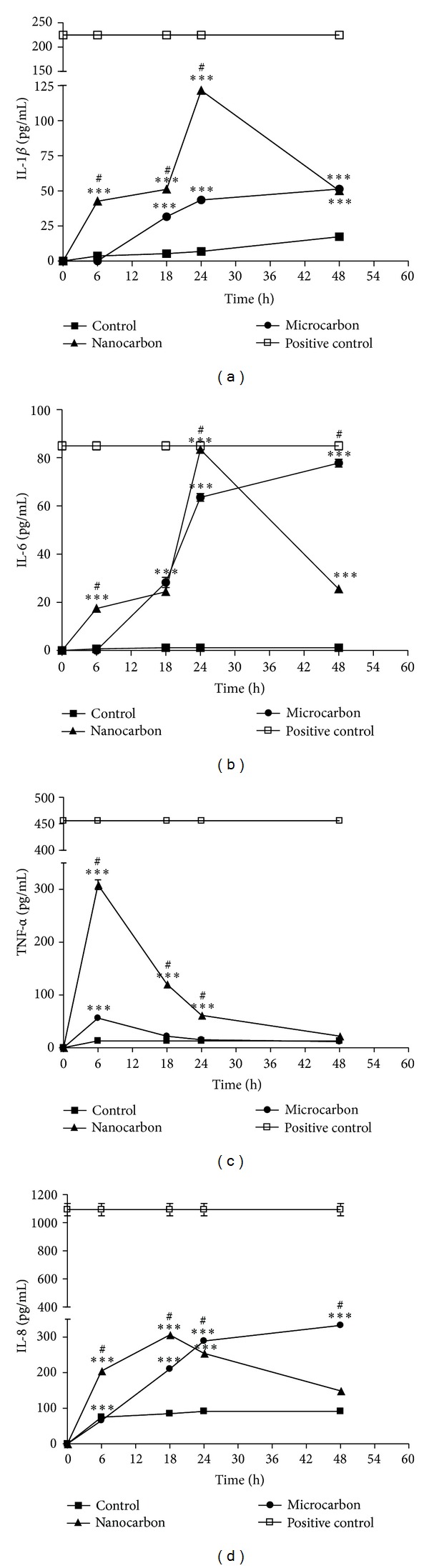
Proinflammatory cytokine and chemokine level in THP-1 cells exposed to carbon particles at their TC-50 concentration (nano carbon: 591.4 *μ*g/mL and micro carbon 687.1 *μ*g/mL) for different times (0, 6, 18, 24, and 48 h) as determined by ELISA. A positive control for each assay was run in parallel as per manufacturer's instruction. (a) IL-1*β* level, (b) IL-6 level, (c) TNF-*α* level, and (d) IL-8 level. Results are presented as mean ± SEM of three independent replicate experiments. Significance is indicated by ***P < 0.001 versus control. ^#^ *P* < 0.001 indicates significant difference between the particle sizes.

Monocytes secrete inflammatory mediators like cytokines upon stimulation by various agents. The cellular release of the proinflammatory cytokine (IL-1*β*, TNF-*α*, and IL-6) and chemokine (IL-8) into the culture medium was measured when monocytes were exposed to TC-50 concentration of CB particle at different time points (6, 18, 24, and 48 h) (Figure 6). Carbon particles showed significant size- and time-dependent release of IL-1*β*, IL-6, TNF-*α*, and IL-8 from THP-1 cells (Figures 6(a), 6(b), 6(c), and 6(d)). In case of nano-CB particles, there was time-dependent release of cytokine up to 24 h which declined thereafter while it remained increasing up to 48 h in case of micro-CB particles. The nanosized particles were more potent than the larger particles in inducing the release of all the cytokines (*P* < 0.001).

### 3.5. MCP-1 and COX-2 mRNA Expression

**Figure 7:**
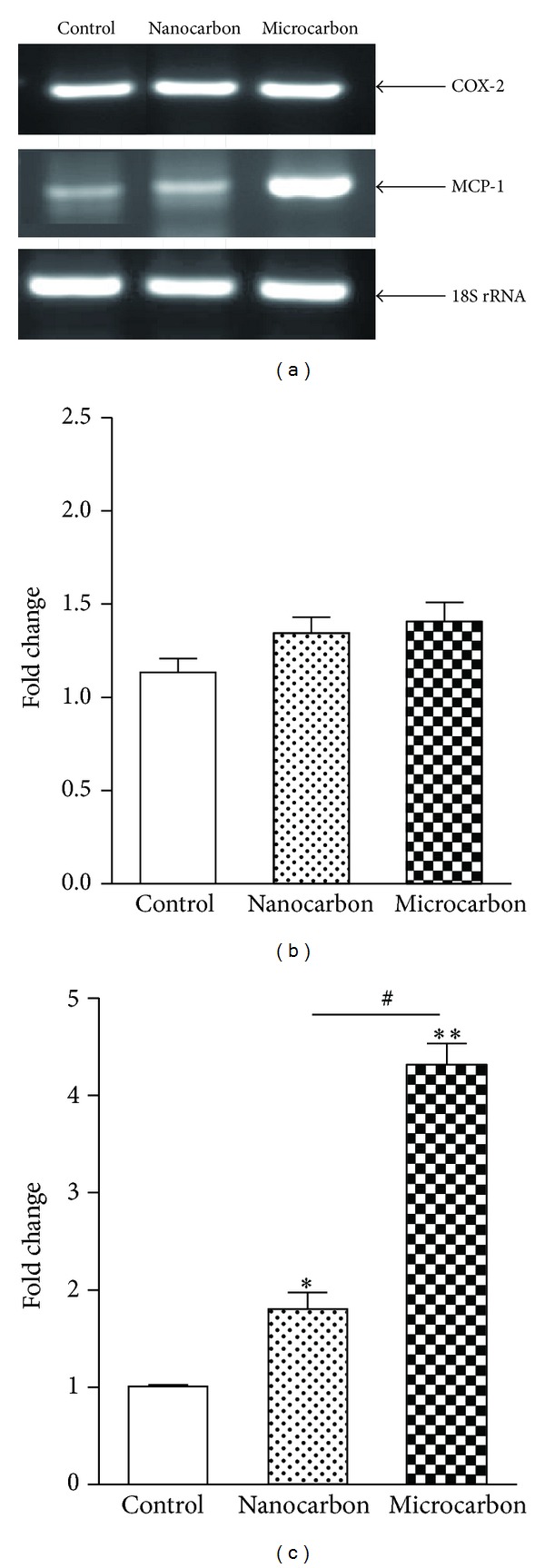
Effects of carbon particles (nano and micro) on mRNA levels of COX-2 and MCP-1 gene. THP-1 cells were exposed to carbon particles at their TC-50 concentration (nano carbon: 591.4 *μ*g/mL and micro carbon 687.1 *μ*g/mL). (a) COX-2 and MCP-1 gene expression on agarose gel. Gene expressions are analyzed by densitometric analysis using Image J software. Results are also expressed as a fold change over the control group. (b) Densitometric bar diagram of COX-2 gene. (c) Densitometric bar diagram of MCP-1 gene. Statistically significant differences were assessed as compared to the controls (*P < 0.05 and **P < 0.01). ^#^ *P* < 0.001 indicates significant difference between the particle size.

A significant upregulation of MCP-1 gene expression was observed with both sizes of CB particles (Figure 7(a)). Micron size of CB particles exhibited more expression of MCP-1 gene than nanosize (Figure 7(c)). However, no significant change was observed in COX-2 gene expression by both sizes of CB particles (Figures 7(a) and 7(b)) as compared to control.

### 3.6. DNA Analysis of THP-1 Cells

**Figure 8:**
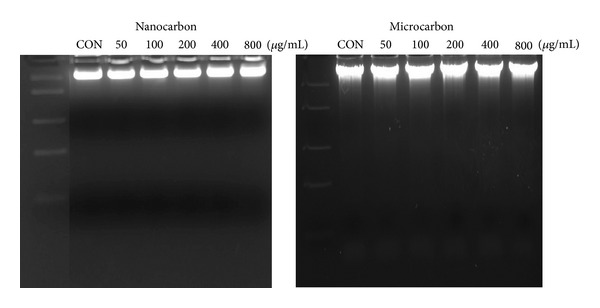
Effect of carbon particles (nano and micro) on DNA of THP-1 cells. The cells were exposed to 50, 100, 200, 400, and 800 *μ*g/mL of carbon particles for 24 h. Equal amount of DNA (20 *μ*g) was loaded on agarose gel and visualized under UV light.

DNA damage was studied by observing smearing pattern on agarose gel under UV light. Exposure of THP-1 cells to both sizes of CB particles for 24 h did not exhibit any smearing pattern on agarose gel (Figure 8).

## 4. Discussion

The inconsistencies of the effects of different CB samples in relation to carcinogenicity and toxicity were reported [23, 24]. These inconsistencies are likely to be related to variation in the particle sizes between the different samples. Therefore, the present in vitro study compared the potential differences in the ability of nano- and micro-CB particles to produce toxicity on exposure to human monocytes, THP-1. The physicochemical characteristics of nano- and micron size of CB particles were extensively characterized. The average size of the particles was in agreement with the size provided by the supplier. DLS analysis revealed a hydrodynamic size of dispersed particles in cultured medium indicating agglomeration in aqueous media which was further corroborated by zeta potential measurement. Needless to surprise, the mean particle sizes and size distribution of particles (measured by DLS) were found enhanced when measured in aqueous media compared to measurements in the dry phase (measured by TEM). To minimize the effects of particles aggregation and sedimentation, suspension of particles was always freshly prepared and sonicated before each experiment.

In this study, the cytotoxicity results demonstrated that nanoparticles of CB were more potent than its micron size in causing toxicity to THP-1 cells as revealed by MTT assay. Both sizes of particles followed concentration-dependent toxicity in monocytes. The effect of CB particle on phagocytic ability of monocytes was also demonstrated. It was found that only nano-CB particle impaired the phagocytic capacity of monocytes. Our findings of impaired phagocytosis in nano-CB particle exposed cells support the findings of Renwick et al. who demonstrated that ultrafine (CB and TiO_2_) particles impaired macrophage phagocytosis to a greater extent than fine particles compared on a mass basis [25].

The study of inflammatory responses of THP-1 cells on exposure to nano- and micro particles of CB revealed the induction of proinflammatory cytokines IL-1*β*, IL-6, and TNF-*α*. Many cytokines including IL-1, IL-6, and TNF-*α* activate functions of inflammatory cells during acute inflammatory responses. These cytokines increase the vascular permeability and thus cause swelling and redness associated with inflammation. IL-1 and IL-6 are responsible for fever reactions while TNF-*α* stimulates endothelial cells and is responsible for hypotension [26]. The nanoform of CB particle showed increase in release of IL-1*β* and IL-6 with increasing time of exposure up to 24 h and then declined thereafter distinctly from its micron form. TNF-*α* secretion was induced at early time point, that is, at 6 h, which later on decreased with time. It was observed that the secretion of IL-1*β*, IL-6, and TNF-*α* cytokines was more profound with nanosize than with micron size. This suggests that the response of particles to proinflammatory cytokine release was size dependent. Chemokines are secondary proinflammatory mediators; that is, they are typically induced by primary proinflammatory mediators such as IL-1 or TNF. In the present study, gene expression of MCP-1, a CC chemokine, and release of IL-8, a member of the CXC chemokine subfamily, were studied. MCP-1 stimulates both chemotaxis of monocytes and several cellular events associated with chemotaxis. The result of the present study demonstrated the stimulation of chemotaxis as revealed by upregulation of MCP-1 gene and increased release of IL-8 on exposure to CB particles. Niwa et al. [27] have also shown the upregulation of IL-6 and MCP-1 in rats after inhalation exposure of CB and substantiate the findings. IL-8 is a chemokine which plays a key role in the activation of neutrophils and their recruitment to the site of inflammation [28]. Results demonstrated increase in the release of IL-8 with time after exposure to both sizes of CB particles. Kim et al. [29] reported increased expression of IL-8 mRNA and protein on exposure of ultrafine carbon particles in normal human bronchial epithelial cells and supported the findings. Noticeably, the MCP-1 gene expression was more upregulated by micron size of CB than nanosize. Similar results were observed with another chemokine, IL-8, where micron size of CB was able to induce more secretion of IL-8 at later time of exposure. Another inflammatory marker, COX-2, was also studied. COX-2, an inducible isoform of cyclooxygenase, is induced by several mitogenic and proinflammatory stimuli including LPS, interleukin-1 (IL-1*α* and IL-1*β*), and TNF-*α* [30, 31]. In the present study, CB particles did not show any change in the COX-2 expression. Overall, in accordance with the results of Ferin et al. [32] and Li et al. [33], it was found that nano-CB induced a greater toxicity and inflammatory response than micro-CB in human monocytes.

In the present study, the effect of CB particles on DNA damage of human monocytes was also studied. In assessing toxicity, DNA damage to macrophages is an important outcome since (i) these cells remove inhaled NPs [34] and (ii) DNA damage is considered to be an important initial event in various diseases including carcinogenesis [35]. The results showed that there was no smearing pattern observed on agarose gel indicating that both sizes of CB particles did not cause any DNA damage.

In conclusion, a comparative toxicity study between nano- and micro-CB particle resulted in size-dependent cytotoxicity and increased inflammatory responses in human monocytes, THP-1 cells. Nano-CB particles altered the phagocytic capacity of monocytes although micro-CB had no significant effect on phagocytic capacity of monocytes. However, both sizes of CB particle did not have any effect on DNA of THP-1 cells. Further studies are required to elucidate the exact pathway of inflammatory response induced by CB particles in immune cells.

## Figures and Tables

**Table 1:** Primer sequence of cyclooxygenase-2 (COX-2), monocyte chemoattractant protein-1 (MCP-1), and 18S rRNA gene used in this study.

Primer name	Primer sequences	*T*_*m*_	Number of cycles performed
COX-2	F: TTCAAATGAGATTGTGGGAAAATT	59.1	35
	R: AGATCATCTCTGCCTGAGTATCTT		

MCP-1	F: AATCAATGCCCCAGTCACCTGC	62.1	35
	R: CGCAGTTTGGGTTTGCTTGTCC		

18S rRNA	F: GTAACCCGTTGAACCCCATT	58.3	35
	R: CCATCCAATCGGTAGTAGCG		

**Table 2:** Particle characterization.

Particles	Description	Average size^a^	Size using TEM^b^ (nm)	Size in media^c^ (nm)	PDI^d^	Zeta potential^e^ (mV)
Nanocarbon	Carbon nanopowder	<50 nm	53.7 ± 10.2	235.5 ± 24.9	0.005 ± 0.001	−39.9 ± 4.91
Microcarbon	Carbon	~500 nm	524.9 ± 8.1	636.7 ± 17.2	0.074 ± 0.008	−15.6 ± 6.37

^a^According to the manufacturer Sigma-Aldrich.

^b^Using transmission electron microscopy.

^c,d, and e^Using ZetaPALS.

PDI: polydispersity index.

**Table 3:** Calculated TC-50 concentrations.

Cell type	Carbon particles	
	Nanocarbon	Microcarbon
THP-1	591.4 µg/mL	687.1 µg/mL
	(490.0 to 713.6)	(506.9 to 931.3)
